# The Chinese herbal formula pediatric Anshen Bunao Granules for the treatment of tic disorder: study protocol for a randomized, double-blind, active-controlled trial

**DOI:** 10.3389/fneur.2026.1822528

**Published:** 2026-05-19

**Authors:** Wenjing Liu, Yuchao Luo, Yuge Cao, Hui Zhang, Yiwei Chen, Ting Zhang, Yu Liu

**Affiliations:** 1 The Fourth Clinical Medical College of Guangzhou University of Chinese Medicine, Shenzhen, Guangdong, China; 2 Shenzhen Traditional Chinese Medicine Hospital, Shenzhen, Guangdong, China

**Keywords:** Chinese herbal medicine, hospital-prepared traditional Chinese medicine formulation, pediatric Anshen Bunao Granules, randomized controlled trial, tic disorder, Tourette syndrome

## Abstract

**Background:**

Tic disorder (TD) affects 2.68% of children in China. It is classified as a movement disorder, with dysfunction in cortical–striatal–thalamic–cortical circuits implicated in its pathophysiology. Western medical treatments for TD may be associated with adverse reactions and suboptimal adherence in some patients. Pediatric Anshen Bunao Granules (PABG) is an in-house traditional Chinese medicine (TCM) preparation with over 20 years of clinical use for TD, approved by the Guangdong Provincial Drug Administration for use in medical institutions across Guangdong Province. Observational studies have reported effects of PABG; however, high-level evidence remains lacking. This study aims to evaluate the efficacy and safety of PABG in children with TD.

**Methods:**

This is a randomized, double-blind, active-controlled, parallel-group trial. A total of 150 children aged 4–16 years with TD (TCM syndrome: Spleen Deficiency with Phlegm Accumulation and Wind-Phlegm Disturbance Syndrome) will be recruited from Shenzhen Traditional Chinese Medicine Hospital, Guangdong Province, China. Eligible participants will be stratified by age group and baseline Yale Global Tic Severity Scale (YGTSS) total score, and randomly allocated at a ratio of 2:2:1 to the low-dose PABG group, high-dose PABG group, or active control group (receiving 5% of the active ingredients to maintain blinding) for 8 consecutive weeks of treatment. Primary outcomes will be the change in YGTSS Total Tic Score (YGTSS-TTS) from baseline to Week 8, and the proportion of participants with a ≥ 30% reduction in YGTSS-TTS at Week 8. Secondary outcomes will be assessed using the YGTSS, the TCM Syndrome Rating Scale (TCM-SRS), and the Gilles de la Tourette Syndrome-Quality of Life Scale for Children and Adolescents (C&A-GTS-QoL). Safety assessments will include vital signs, physical examination, laboratory tests, ECG, and adverse events.

**Discussion:**

This trial will provide evidence on the efficacy and safety of PABG for children with TD. The findings may inform clinical application and potential registration of this in-house TCM preparation.

**Clinical trial registration:**

https://itmctr.ccebtcm.org.cn/, ITMCTR2025001659.

## 1 Introduction

Tic disorder (TD) is a neuropsychiatric condition primarily manifesting as rapid and involuntary motor and/or vocal tics, typically emerging during childhood and adolescence (1). It frequently co-occurs with multiple mental and/or behavioral disorders, such as Attention Deficit Hyperactivity Disorder (ADHD), Obsessive-Compulsive Behavioral /Disorder (OCB/OCD), anxiety disorders, depressive disorders, and sleep disorders (2). Motor tics manifest as involuntary muscle contractions, whereas vocal tics present as abnormal vocalizations; certain triggers, such as emotional fluctuations or dietary irregularities, may exacerbate symptoms. According to the diagnostic criteria of the Diagnostic and Statistical Manual of Mental Disorders, Fifth Edition (DSM-5) (3), TD is categorized into three types: transient tic disorder (TTD), chronic motor or vocal tic disorder (CTD), and Tourette syndrome (TS). The mean age at onset for TD is 5 years, with peak symptom severity occurring between 8 and 12 years of age (4). A 2022 study by Jafari et al. (5) indicated a global prevalence of TS of 0.5%. Research published in 2023 indicates an overall TD prevalence of 2.68% in China (6), with males exhibiting a higher incidence of severe symptoms than females (7). Tics can impair patients’ daily functional activities, leading to social isolation, interpersonal conflicts, peer bullying, and disruption to academic or occupational pursuits, thereby diminishing quality of life and adversely affecting the healthy development of personality and psychological well-being (8).

The precise etiology and pathogenesis of TD are widely recognized as involving multifactorial mechanisms, including neurotransmitter dysregulation, genetics, immunological factors, trace element imbalances, and socio-environmental and psychological influences (9, 10). Recent evidence using the 2D:4D digit ratio, a marker of fetal sex hormone exposure, has also suggested a link between these hormone levels and tic disorder (11). Among these, dysfunction within cortical–striatal–thalamic–cortical (CSTC) circuits has emerged as a key neurobiological substrate underlying the pathophysiology of tic disorders (12, 13). The proposed mechanism involves aberrant neuronal firing in the striatum, which disrupts its inhibitory output to the thalamus. Loss of this inhibition allows increased thalamic drive to the sensorimotor cortex, resulting in excessive cortical excitability that produces motor and vocal tics (14, 15). Regarding the treatment of pediatric TD, guidelines issued by the European Society for the Study of Tic Disorders (ESSTS) and the American Academy of Neurology (AAN) recommend psychological interventions and/or pharmacological treatment (16–18). The antipsychotic drug aripiprazole is the most commonly prescribed medication in European clinical practice and has been shown to effectively improve tic symptoms (19). Commonly used antipsychotic drugs include central *α*-adrenergic receptor agonists, selective 5-hydroxytryptamine reuptake inhibitors, and dopamine receptor antagonists. While these medications can alleviate tic symptoms, they present challenges such as a slow onset of action, prolonged treatment duration, multiple adverse effects, and high relapse rates. Certain drugs may suppress normal central nervous system excitability, leading to reduced medication adherence among children and parents (20, 21).

TD treatment necessitates the exploration of additional safe and effective therapeutic approaches to enhance children’s quality of life. Modern clinical practice in traditional Chinese medicine (TCM) (22) demonstrates its advantages in syndrome differentiation–based treatment for childhood TD: it effectively alleviates tic symptoms and comorbidities, reduces recurrence rates, and is associated with fewer adverse reactions. In addition, herbal formulations, such as 5-ling Granules and Ningdong Granules, have been included in the list of compounds with moderate-certainty evidence of therapeutic efficacy (19). Consequently, TCM has emerged as a complementary clinical treatment option for childhood TD (23). TCM possesses millennia of accumulated clinical experience regarding the understanding and management of childhood TD, attributing its onset to factors such as the child’s emotional state and nutritional status (24, 25). Based on fundamental TCM theory, TD can be categorized into several syndromes, with the following four being the most common in clinical practice: Qi Stagnation Transforming into Fire Syndrome, Spleen Deficiency with Phlegm Accumulation and Wind-Phlegm Disturbance Syndrome (SDPWS), Spleen Deficiency with Liver Hyperactivity Syndrome, and Yin Deficiency Stirring Wind Syndrome (26). SDPWS is the most common pattern, accounting for nearly half of the cases (27). Clinically, SDPWS typically presents with motor tics (e.g., eye blinking, head nodding, shoulder shrugging) along with signs of spleen deficiency (e.g., poor appetite, sallow complexion, restless sleep). In contrast, the other patterns show distinct features: Qi Stagnation Transforming into Fire Syndrome is characterized by forceful, frequent tics, irritability, and red face; Spleen Deficiency with Liver Hyperactivity Syndrome presents with weak, intermittent tics, fatigue, and chest distension; Yin Deficiency Stirring Wind Syndrome manifests as chronic, low-intensity tics, night restlessness, and dry stools.

Pediatric Anshen Bunao Granules (PABG) is a pediatric traditional Chinese medicine formulation developed by Shenzhen Hospital of Traditional Chinese Medicine, Guangdong Province, China. Its formula is derived from a modification of Ditan Decoction, a classic phlegm-resolving TCM formula. Registered as an in-house TCM preparation at Shenzhen Hospital of Traditional Chinese Medicine in 2001, it was approved by the Guangdong Provincial Drug Administration for clinical use within medical institutions across Guangdong Province. Over the past 5 years, cumulative usage has reached 64,000 boxes (10 boxes per course), serving over 6,400 pediatric patients. Preliminary pharmacodynamic studies indicate that PABG exhibits antioxidant properties, protects against neuronal damage, and exerts neurotrophic effects (28). A small-scale clinical trial utilized PABG as the intervention and haloperidol tablets (a second-line treatment for TD [29]) as the control; efficacy was assessed using the Yale Global Tic Severity Scale (YGTSS). Results demonstrated that after 6 months of treatment, the PABG group exhibited superior overall and long-term efficacy compared to the control group, alongside a lower disease recurrence rate (29). This formulation comprises 18 Chinese medicinal ingredients, including Acorus tatarinowii, Arisaema cum bile, Polygalae radix, Lapis chloriti, Astragali radix, Pinellia ternata, Poria cocos, Citri reticulatae pericarpium, Haliotidis concha, Notopterygii rhizoma et radix, Corni fructus, Alpiniae oxyphyllae fructus, Aurantii fructus, Fructus tritici levis, Hordei fructus germinatus, Massa Medicata Fermentata, Crataegi fructus, and Glycyrrhizae radix et rhizoma.

Current pharmacological research indicates that multiple herbs in the formulation possess distinct neuropsychiatric–related therapeutic effects: Acorus tatarinowii modulates synaptic plasticity by regulating dopaminergic and glutamatergic neurotransmission, thereby potentially intervening in TD (30); Arisaema cum bile inhibits neuroinflammation and modulates the farnesoid X receptor (FXR) and gamma-aminobutyric acid (GABA) signaling pathways, thereby alleviating convulsions triggered by febrile seizures (31); Polygalae radix has been reported to exhibit sedative, antioxidant, anti-neuroinflammatory, and neuroprotective effects (32); Lapis chloriti has been traditionally employed to relieve epileptic symptoms (33); and Astragali radix possesses anti-inflammatory, antioxidant, and neuroprotective properties (34). Convulsions constitute a core clinical manifestation of TD, sharing common pathophysiological mechanisms with febrile seizures and epilepsy; sedative and neuroprotective actions are closely associated with the regulation of TD symptoms. Furthermore, although the pathogenesis of TD remains incompletely understood, research suggests that neuroinflammation and oxidative stress may contribute to its pathological process (35, 36). The anti-inflammatory and antioxidant properties of Astragali radix may offer potential therapeutic relevance for TD management. The experimental evidence for these Chinese medicinal herbs provides direct or indirect mechanistic support for the clinical application of PABG in alleviating childhood TD symptoms.

In recent years, the National Medical Products Administration of China (NMPA) has introduced multiple policies and regulations encouraging the inheritance and innovative development of TCM. For instance, Article 24 of the Special Provisions on the Registration Management of Traditional Chinese Medicines stipulates that, when conducting the clinical development of TCM with established human experience, real-world evidence may be used as one of the supporting bases for product market authorization under specific conditions. This study, guided by documents such as the Classification of Traditional Chinese Medicine Registration and Requirements for Submission Materials (37) issued by the NMPA in 2020 and the Technical Guidelines for Clinical Trial Design and Evaluation of Traditional Chinese Medicine for Tic Disorders (2021 Edition) (38) published by the Clinical Pharmacology Branch of the Chinese Association of Chinese Medicine (CACM), proposes to conduct a clinical trial of PABG. This trial will generate evidence to support the design of subsequent confirmatory trials and facilitate potential market authorization of this in-house TCM preparation.

## 2 Methods

### 2.1 Aim

This randomized controlled trial (RCT) aims to evaluate the efficacy and safety of two dose levels of PABG (low and high) compared with a minimal-dose active control (5% PABG). The dose-gradient design allows for exploratory assessment of the dose–response relationship to inform future dose selection.

### 2.2 Setting

This is a randomized, double-blind, active-controlled, parallel-group clinical trial. It will be conducted at Shenzhen Traditional Chinese Medicine Hospital. The trial’s design and conduction will adhere to the Declaration of Helsinki and have been approved by the ethics committees at Shenzhen Traditional Chinese Medicine Hospital (No. K2025-111-02). The trial was registered at International Traditional Medicine Clinical Trial Registry (No. ITMCTR2025001659) on 3 September 2025.

### 2.3 Recruitment

A total of 150 participants will be recruited from Shenzhen Traditional Chinese Medicine Hospital.

### 2.4 Inclusion criteria

Participants must meet all of the following criteria to be eligible for enrollment:

1. Following the exclusion of other potential diseases via electroencephalogram, psychological testing, and laboratory assessments, a diagnosis of Transient Tic Disorder (TTD), Chronic Tic Disorder (CTD), or Tourette Syndrome (TS) is confirmed according to the tic disorder diagnostic criteria in the Diagnostic and Statistical Manual of Mental Disorders, Fifth Edition (DSM-5) (American Psychiatric Association, 2013) (3).
2. The traditional Chinese medicine (TCM) syndrome differentiation is characterized as Spleen Deficiency with Phlegm Accumulation and Wind-Phlegm Disturbance Syndrome (SDPWS) (Table 1).
3. Aged 4–16 years (inclusive), regardless of gender.
4. A total score of ≥ 25 on the Yale Global Tic Severity Scale (YGTSS).
5. For participants aged ≥ 8 years, the ICF must be jointly signed by the participant and their legal guardian; for participants aged < 8 years, the ICF must be signed by the legal guardian alone.

**Table 1:** Diagnostic criteria for SDPWS.

Symptom type	Symptoms
Main symptom	(1) Frown and wink; (2) Open mouth and grin; (3) Shake head and shrug shoulders; (4) Shake hands and kick legs; (5) Utter foul language
Secondary symptom	(1) Irritability; (2) Poor sleep quality; (3) Sallow complexion; (4) Dark periorbital discoloration; (5) Sticky or dry stools

The diagnostic criteria are formulated based on the official guidelines issued by the Pediatric Branch of the Chinese Association of Chinese Medicine (2013). Syndrome differentiation is confirmed if a participant presents at least one main symptom plus two to three secondary symptoms. Tongue and pulse manifestations are regarded as supplementary reference indicators.

### 2.5 Exclusion criteria

Participants will be excluded if they meet any of the following:

1. TTD with a disease course ≤ 90 days (TTD with a course > 90 days is eligible).
2. Involuntary movements attributable to other conditions (e.g., rheumatic chorea, Huntington’s disease, Wilson’s disease, choreoathetosis, myoclonus, acute motor disorders, hysterical spasms, epilepsy, drug-induced extrapyramidal disorders, substance/drug-induced motor or muscle tone disorders).
3. Comorbidities including Attention Deficit Hyperactivity Disorder (ADHD), Obsessive-Compulsive Behavior/Disorder (OCB/OCD), learning disorders, sleep disorders, mood disorders, or self-injurious behavior.
4. Confirmed diagnosis of depression or schizophrenia at screening.
5. Use of medications indicated for tic disorder (TD) treatment (or effective for tics) within 14 days prior to randomization (e.g., haloperidol, Jiuwei Xifeng Granules).
6. History of adequate, standardized treatment with ≥ 2 anti-TD drugs (different mechanisms of action) for > 1 year, with no significant efficacy and persistent symptoms.
7. Current receipt of behavioral therapy (eligible after a washout period).
8. Participation (current or past 90 days) in other drug clinical trials, or within 5 half-lives of the study drug (whichever is longer).
9. Hypersensitivity to the study drug or its excipients.
10. Severe comorbidities (heart, brain, liver, kidney, or hematopoietic system); or serum levels of aspartate aminotransferase (AST)/alanine aminotransferase (ALT) > 2.5 × upper limit of normal (ULN); or alkaline phosphatase (ALP)/total bilirubin > 2 × ULN; or clinically significant electrocardiogram (ECG) abnormalities (per investigator assessment).
11. Other conditions deemed unsuitable for enrollment (per investigator judgment).

### 2.6 Trial design

**Figure 1:**
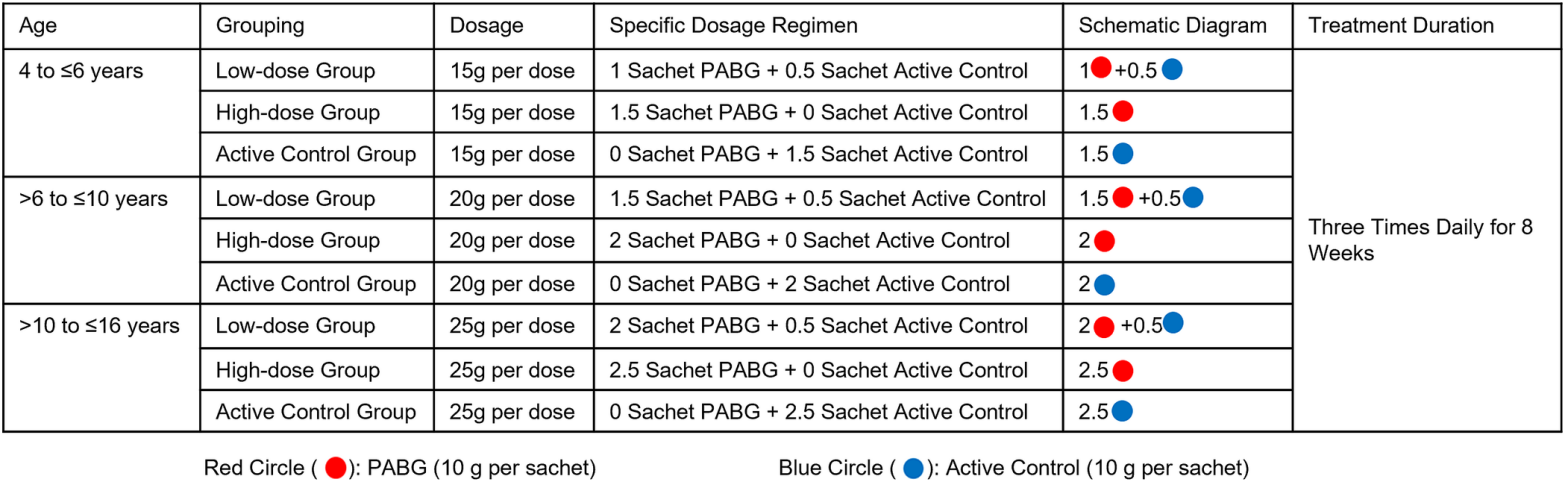
Dosage and administration regimen of PABG and active control according to age groups and treatment groups.

This study will comprise three phases as detailed below:

1. Screening period (W-2 to W0): Participants and their legal guardians will enter the screening phase after signing the Informed Consent Form (ICF), which has been approved by the Medical Ethics Committee of Shenzhen Traditional Chinese Medicine Hospital. Investigators will provide detailed explanations of the study objectives, treatment methods, procedures, potential benefits, and risks, as well as guidance on medication administration and lifestyle precautions during the study period.
2. Treatment period (W1–W8): On Day −1 (D-1), participants who meet all inclusion criteria and none of the exclusion criteria will undergo final eligibility confirmation. Baseline assessments will be performed, including the YGTSS, TCM Syndrome Rating Scale (TCM-SRS), Gilles de la Tourette Syndrome–Quality of Life Scale for Children and Adolescents (C&A-GTS-QoL), and safety evaluations. Eligible participants will then be allocated to the low-dose PABG group, high-dose PABG group, or active control group (containing 5% of the active ingredients for blinding) in a 2:2:1 ratio using stratified block randomization with age and disease severity as stratification factors. According to their group assignment and age, participants will receive the corresponding dosage of PABG or active control, three times daily for 8 consecutive weeks. The detailed dosage regimen is presented in Figure 1. Investigators will conduct follow-up visits at Weeks 2, 4, 6, and 8 after treatment initiation (±2 days permitted). Participants and their legal guardians will document symptom episodes and complete efficacy assessments and safety examinations in accordance with the protocol.
3. Follow-up Period (W12): Participants will return to the study center 4 weeks after the end of treatment (±2 days permitted), at which point investigators will collect efficacy and safety evaluation data.

**Figure 2:**
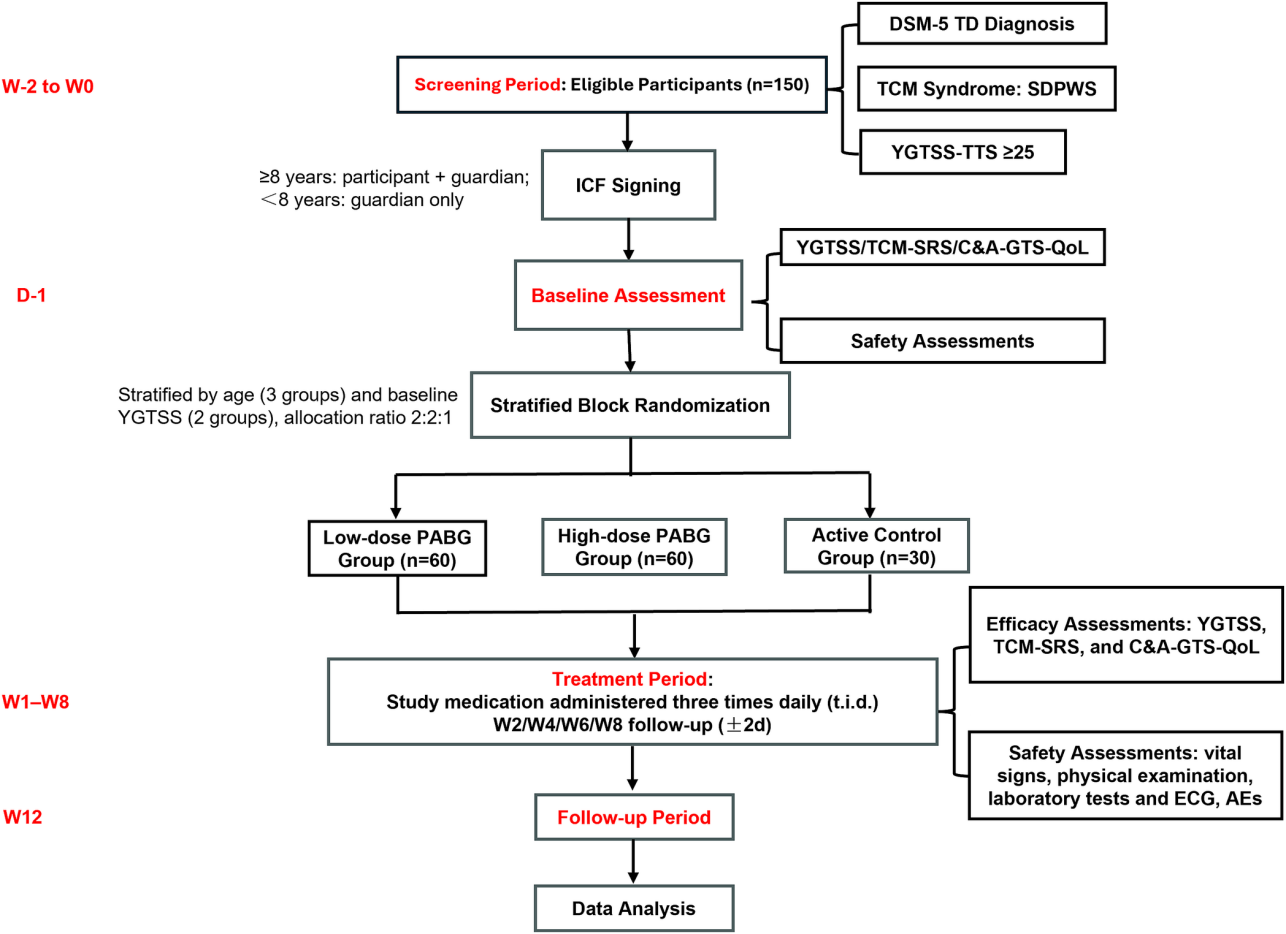
The flowchart of the trial.

The trial flow diagram is illustrated in Figure 2.

### 2.7 Sample size

To date, no active-controlled trial has evaluated PABG for the treatment of tic disorders with SDPWS. The primary endpoint of this study is the change from baseline in the YGTSS–Tic Severity Score (YGTSS-TTS) at Week 8. Preliminary data from our pilot study indicated a mean baseline YGTSS-TTS score of 22.8. The minimal clinically important difference (MCID) for the YGTSS-TTS is defined as a ≥ 25% reduction from baseline (39), corresponding to 5.7 points. Based on effect sizes reported in clinical trials of comparable Chinese patent medicines for tic disorders (40), and considering the dose-gradient design of the present study, we assume a mean reduction of 8.2 ± 7.0 points in the active control group, 12.4 ± 6.8 points in the low-dose group, and 12.9 ± 6.6 points in the high-dose group.

With a two-sided significance level of *α* = 0.025 (Bonferroni correction for two comparisons: high-dose vs. active control and low-dose vs. active control) and *β* = 0.20 (80% power), and an allocation ratio of 2:2:1, the sample size was calculated using PASS version 20.0 (NCSS, LLC, Kaysville, UT, USA). Allowing for a 15% dropout rate, the adjusted and rounded total sample size was determined to be 150 participants, with 60 in the high-dose group, 60 in the low-dose group, and 30 in the active control group. The primary comparisons are each active dose versus the active control; the sample size was not powered to detect a statistically significant difference between the two active doses. The close projected outcomes (0.5-point difference) reflect a conservative assumption, and the dose–response assessment is exploratory.

The assumed effect size between each active dose and the active control (approximately 4.2 to 4.7 points) is consistent with the effect observed in a previous trial of a similar Chinese patent medicine (40), where the active product showed a 4.8 point greater reduction in YGTSS-TTS compared with placebo at week 8. Although this effect is slightly below the MCID of 5.7 points, as an exploratory dose-finding trial, any statistically significant signal, even if below the MCID, would support further investigation in a larger confirmatory trial. The MCID will be used for clinical interpretation of the final results.

### 2.8 Participants information

Information collected during the screening period will include demographic data (date of birth, gender, ethnicity); TD-related information, including the date of initial diagnosis, date of first medical consultation for TD-related symptoms, frequency and type of past symptoms, disease subtype (TTD, CTD, TS), and a history of prior pharmacological and non-pharmacological treatments for TD; as well as medical history unrelated to TD, a history of pharmacological and non-pharmacological treatments within the 3 months prior to screening, and allergy history.

### 2.9 Preparation of study medications

PABG (Batch No.: Yue Z20070083) and the active control used in this study will be uniformly prepared and packaged by the Shenzhen Traditional Chinese Medicine Preparation Center. Both will be verified as compliant prior to use, with each sachet containing 10 g. The active control herbal preparation has been validated in previous studies (41). The ingredients and quantities for PABG are presented in Table 2. All herbal ingredients will be sourced from government-authorized TCM suppliers. Each herb will undergo testing for microbiological contamination, heavy metals, and pesticide residues to ensure compliance with Chinese regulatory requirements, with the entire quality assessment process overseen by senior TCM practitioners.

**Table 2:** Ingredients and quantities for 100 sachets (1,000 g) of PABG.

Herbal name (Latin & Vernacular)	Quantity (g)
Acorus tatarinowii (Shi-Chang-Pu)	250
Poria cocos (Fu-Ling)	165
Fructus Tritici levis (Fu-Xiao-Mai)	250
Astragali radix (Huang-Qi)	165
Hordei fructus Germinatus (Mai-Ya)	250
Citri reticulatae pericarpium (Chen-Pi)	85
Arisaema cum bile (Dan-Nan-Xing)	165
Corni fructus (Shan-Zhu-Yu)	165
Lapis chloriti (Qing-Meng-Shi)	250
Notopterygii rhizoma et radix (Qiang-Huo)	85
Massa Medicata Fermentata (Liu-Shen-Qu)	165
Polygalae radix (Yuan-Zhi)	165
Aurantii fructus (Zhi-Qiao)	165
Alpiniae oxyphyllae fructus (Yi-Zhi-Ren)	165
Crataegi fructus (Jing-Shan-Zha)	165
Glycyrrhizae radix et rhizoma (Gan-Cao)	165
Pinellia Ternata (Qing-Ban-Xia)	165
Haliotidis concha (Shi-Jue-Ming)	250
Sucrose	680
Dextrin	227
In total	4,142

The 18 Chinese herbal decoction pieces in the prescription will be decocted twice in water, each time for 2 h. The decoction liquids will be combined and filtered, and the filtrate will be concentrated to a clear paste with a relative density of 1.25–1.30 (measured at 80 °C). After cooling, 1,500 mL of ethanol will be added, stirred thoroughly, and allowed to stand for 24 h. The supernatant will be decanted to recover ethanol and then reconcentrated to a clear paste with a relative density of 1.25–1.30 (measured at 80 °C). Sucrose powder and dextrin will be added, mixed uniformly, granulated, and dried at 65–75 °C to produce 1,000 g. The raw material–to–product weight ratio in this process is approximately 4:1. The active control preparation contains 5% of the active ingredients of PABG, termed the minimal-dose active control, with caramel color and chocolate brown pigment added in an 8:3 ratio to ensure a consistent appearance, taste, weight, and smell with PABG. The formulation center will uniformly package all investigational medicinal products in compliance with relevant Chinese pharmaceutical regulatory requirements, labelling them as “Drug A” and “Drug B” to maintain the double-blind design.

The 5% active ingredient concentration is not intended to produce a pharmacological effect but is necessary to match taste, odor, and appearance for successful blinding. We acknowledge that this active control is not an inert placebo. Therefore, the design compares each PABG dose (low and high) against a low-dose active control. Any treatment effect may be underestimated (biased toward the null). This limitation is further discussed in Section 6.

### 2.10 Randomization and blinding

A stratified block randomization sequence will be constructed using SAS software (version 9.4 or later; SAS Institute Inc., Cary, USA) by a team of randomization statisticians. The stratification is based on two factors: age categories (≥4 to ≤6 years, >6 to ≤10 years, >10 to ≤16 years) and baseline disease severity (YGTSS total score ≥25 to ≤50, >50).

All eligible participants will be randomly allocated to the high-dose PABG group, low-dose PABG group, or active control group at a 2:2:1 ratio via the Interactive Web Response System (IWRS). Authorized investigators will access the system to retrieve the participants’ treatment assignments for randomization. The outcomes of the randomization process will be strictly kept confidential.

Neither the participants nor the research team will be informed of the treatment assignments until the conclusion of the study. Emergency unblinding will be carried out through the IWRS in scenarios such as severe deterioration of participants’ conditions requiring emergency rescue or as mandated by regulatory authorities. The rationale, date, and time of unblinding will be documented in the electronic Case Report Form (eCRF), and the relevant participants will be classified as dropouts.

### 2.11 Concomitant care

Participants must not use other medications for the treatment of TD to avoid interference with PABG efficacy. Concomitant medications and treatments will be documented at each visit from ICF signature through the treatment period, with details recorded in the eCRF. Concomitant treatments for study drug-related adverse events within 4 weeks after the last dose will also be recorded for all subjects who complete treatment or discontinue early.

### 2.12 Monitoring compliance

Participants and their legal guardians will be instructed to record the number of sachets consumed each week. During the treatment period, unused study medication and packaging will be collected at each follow-up visit. The quantity of consumed and returned study medication will be documented in the eCRF to assess medication adherence. A compliance rate of ≥80% will be defined as high adherence.

### 2.13 Discontinuation of study intervention

Participants can voluntarily withdraw from the study at any time and for any reason. All reasons and the time of discontinuation will be documented in the eCRFs and included in the subsequent analysis. Participants will be discontinued from the trial if any of the following events take place: (1) Adverse events of Grade 3 or higher that are considered by the investigator to be related to the study intervention; (2) Serious adverse events that are considered by the investigator to be related to the study intervention; (3) Death; (4) Withdrawal of informed consent by the participant; (5) Termination of the study by the sponsor.

### 2.14 Rescue treatment

If a participant shows inadequate disease control (no improvement in tic symptoms after at least 4 weeks of study medication) and symptoms remain unrelieved after elimination of potential triggers, rescue treatment with tiapride 50 mg daily may be used. The discontinuation time will be determined by the investigator based on subsequent YGTSS scores.

The investigators must document all details of rescue treatment in the eCRF, including the medication name, duration, dosage, and the participant’s response.

## 3 Assessments and endpoints

This study will employ the internationally recognized and widely used Yale Global Tic Severity Scale (YGTSS) (42), which concurrently assesses tic symptoms and social functioning in children with TD. Its core measurement dimensions, abbreviations, and scoring criteria are described as follows: (1) YGTSS–Total Tic Score (YGTSS-TTS): the sum of motor tic scores (0–25 points) and vocal tic scores (0–25 points), with a total score ranging from 0 to 50 points. It quantifies the severity of both motor and vocal tics and reflects the overall tic symptom burden. (2) YGTSS-Impairment Score (YGTSS-IS): ranging from 0 to 50 points, it quantifies the overall impairment of tic symptoms on patients’ daily functioning, covering core domains including social interaction, academic performance, family life, and self-esteem. The YGTSS Total Score is the sum of YGTSS-TTS and YGTSS-IS, with a scoring range of 0–100 points. Severity grading is defined as follows: a YGTSS Total Score <25 points indicate mild severity, 25–50 points indicate moderate severity, and >50 points indicate severe disease (43).

**Figure 3:**
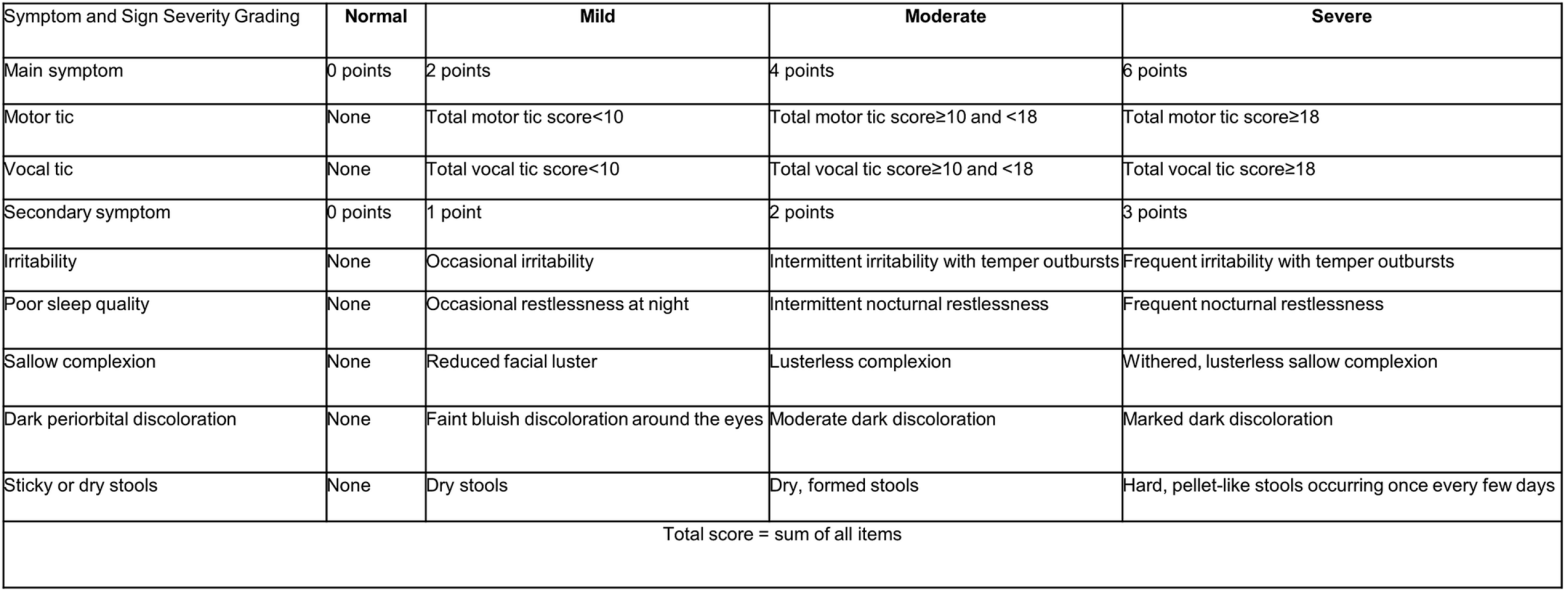
TCM-SRS for tic disorder severity grading.

This study will additionally adopt the TCM-SRS published by the Pediatrics Branch of the Chinese Association of Chinese Medicine (CACM) in 2013, with detailed scoring criteria presented in Figure 3. Concurrently, the C&A-GTS-QoL will be incorporated for efficacy assessment.

**Figure 4:**
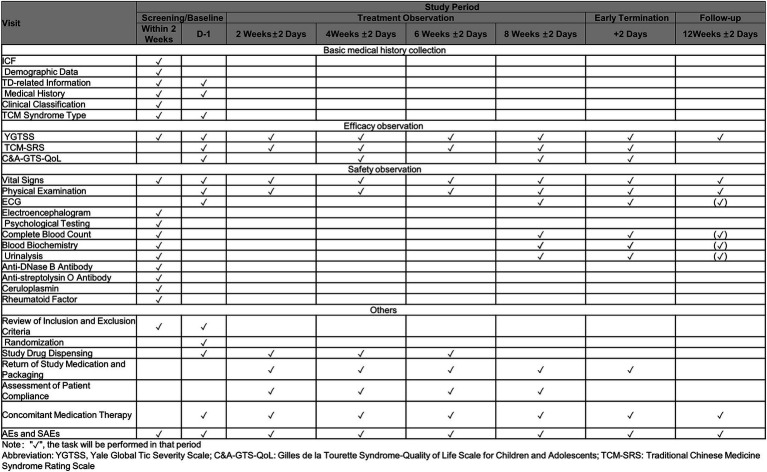
Schedule of trial.

Schedule of study assessments and procedures across all time points is summarized in Figure 4.

### 3.1 Primary efficacy endpoints

The change in YGTSS-TTS from baseline and the proportion of participants achieving a ≥ 30% reduction in YGTSS-TTS, both assessed at Week 8.

### 3.2 Secondary efficacy endpoints

(1) change in YGTSS-TTS from baseline and the proportion of participants achieving a ≥ 30% reduction; (2) percentage change in YGTSS-TTS from baseline and the proportion of participants achieving a ≥ 50% reduction; (3) change in YGTSS Total Score from baseline, percentage change, the proportion of participants achieving a ≥ 30% reduction, and the proportion achieving a ≥ 50% reduction; (4) change in YGTSS-IS from baseline; (5)change in the total TCM-SRS score from baseline, the proportion of participants achieving a ≥ 30% reduction, the proportion achieving a ≥ 50% reduction, and change in individual TCM-SRS symptom scores from baseline; and (6) change in the total C&A-GTS-QoL score from baseline. The specific assessment time points are as follows: assessments for the first endpoint will be performed at Weeks 2, 4, and 6, as well as at 4 weeks post-treatment; assessments for the second, third, and fourth endpoints will be performed at Weeks 2, 4, 6, and 8, as well as at 4 weeks post-treatment; assessments for the fifth endpoint will be performed at Weeks 2, 4, 6, and 8; and assessments for the sixth endpoint will be performed at Weeks 4 and 8.

### 3.3 Safety assessment

Any adverse events (AEs) during the trial will be reported to investigators and documented in the eCRF, including onset time, severity, causality, seriousness, measures taken, and outcome.

All participants will undergo vital signs, physical examination, laboratory tests (complete blood count, blood biochemistry, urinalysis), and ECG.

During screening, anti-streptolysin O antibody, anti-DNase B antibody, ceruloplasmin, and rheumatoid factor will be tested to exclude rheumatic chorea, Wilson’s disease, and other related disorders, with close monitoring of clinically significant abnormalities.

All AEs will be evaluated and analyzed, and their causality with the study intervention will be assessed.

Serious AEs will be reported to the Ethics Committee and the study team within 24 h.

## 4 Statistical analysis

All statistical analyses will be conducted according to a prespecified statistical analysis plan (SAP) by statisticians independent of the research team, who will remain blinded to treatment allocation. A two-sided *P* value <0.05 will be considered statistically significant, and 95% confidence intervals (CIs) will be used to estimate effect sizes. All analyses will be performed using SAS version 9.4 (SAS Institute Inc., Cary, NC, USA).

Continuous variables will be summarized according to their distribution using the number of observations, mean, standard deviation, median, 25th percentile (P25), 75th percentile (P75), and range. Categorical variables will be presented as frequencies and percentages. Demographic characteristics and baseline variables will be summarized descriptively.

For participants who withdraw, drop out, or discontinue treatment, the reasons for discontinuation and the time of the last dose will be recorded. Completed assessment data will be included in the statistical analyses, whereas incomplete data will be treated as missing. Missing data will be handled using multiple imputation methods.

Predefined subgroup analyses for the primary efficacy endpoint will be conducted by gender, age, TD severity and clinical subtype. Sensitivity analyses will include the impacts of prohibited concomitant medications and poor study drug adherence.

### 4.1 Primary outcomes

1. The change from baseline in YGTSS-TTS at Week 8 will be compared between groups using analysis of covariance (ANCOVA), with treatment group, baseline value, and randomization stratification factors included as covariates. Least-squares means, standard errors, and 95% CIs for the change scores, as well as least-squares mean differences with 95% CIs between groups, will be estimated.
2. The proportion of participants achieving a ≥ 30% reduction in YGTSS-TTS from baseline at Week 8 will be calculated with 95% CIs using the Wilson score method. Between-group differences in response rates will be estimated with 95% CIs using the stratified Newcombe method, and group comparisons will be performed using the stratified Cochran–Mantel–Haenszel (CMH) test.

### 4.2 Secondary outcomes

Continuous secondary endpoints will be analyzed using ANCOVA models for between-group comparisons. Categorical secondary endpoints will be analyzed using the same methods as described for Primary Outcome 2.

### 4.3 Safety analysis

AEs will be compared using the chi-square test or Fisher’s exact test, as appropriate. AEs will be coded using the Chinese version of the Medical Dictionary for Regulatory Activities (MedDRA). The severity of AEs and their causal relationship with the investigational drug will be assessed.

### 4.4 Data management and quality control

Before the study initiation, personnel involved in data collection and management will receive training on the study protocol, Good Clinical Practice (GCP), and relevant Standard Operating Procedures (SOPs). A Data Management Plan (DMP) will be developed and revised as needed. An Electronic Data Capture (EDC) system will be used for data entry and management. The data management party will design the eCRF and establish the EDC database per the protocol, which will be put into use after testing and training. Investigators will enter source data accurately and timely, with completed eCRF reviewed by clinical research associates before submission to data managers for verification. Data managers will perform automated and manual verification via the EDC system, feedback queries to investigators for correction, and all data modifications will retain an audit trail. Medical coding will be completed before database locking using MedDRA and WHODrug dictionaries. The sponsor will entrust a Contract Research Organization (CRO) to conduct regular trial monitoring and auditing per SOPs. Laboratories of participating units will establish quality control standards. After data cleaning and verification, the database will be locked with the sponsor’s approval, and post-locking modifications require standardized approval. Relevant trial records will be retained to ensure data traceability and GCP compliance.

## 5 Discussion

To ensure the rationality and scientific validity of the intervention protocol, a comprehensive literature search was conducted on 1 May 2026 in the International Traditional Medicine Clinical Trial Registry (ITMCTR) and PubMed databases to identify clinical studies investigating TCM interventions for childhood TD. The trial design characteristics, inclusion and exclusion criteria, and efficacy evaluation indicators of existing studies were systematically analyzed.

The ITMCTR search for “tic disorder” identified 18 registered studies (excluding our own trial), including 1 observational and 17 interventional studies, of which 11 employed Chinese herbal medicine as the core intervention. Among these 11 studies, only 1 adopted stratified block randomization with study center as the stratification factor. 10 used the YGTSS and TCM syndrome scoring, and 1 included quality-of-life assessments. Concurrently, a PubMed search was performed using the search terms “Tic Disorder OR Transient Tic Disorder OR Chronic Tic Disorder OR Tourette Syndrome”, with filters applied for clinical trials or randomized controlled trials published in the last 10 years. Titles and abstracts were manually screened for studies involving TCM interventions for the treatment of tic disorders in children. After screening, 8 TCM-related studies were identified: 3 investigated non-herbal TCM external therapies (44–46), and 5 evaluated herbal formulas or proprietary Chinese medicines (8, 47–50). All 5 herbal intervention studies used the YGTSS as the primary outcome measure; however, only one applied stratified block randomization, three incorporated TCM syndrome scoring, and none employed standardized quality-of-life scales.

Several methodological limitations were identified in the retrieved studies, including the absence of stratified block randomization with age as a stratification factor and the lack of quality-of-life assessments. In response, the present study will adopt the TCM-SRS in accordance with the Technical Guidelines for Clinical Trial Design and Evaluation of New Traditional Chinese Medicines for Childhood Tic Disorders issued by the Pediatrics Branch of CACM (51), and will implement stratified block randomization based on age and disease severity to reduce the interference of differences in physiological metabolism, drug tolerance, and symptom presentation among children of different ages and disease severities on the study results, enhancing intergroup balance. Furthermore, in line with the recommendations of ESSTS emphasizing functional impairment and quality of life, this study will incorporate the C&A-GTS-QoL (52), thereby establishing a multidimensional efficacy evaluation framework integrating YGTSS, TCM-SRS, and quality-of-life assessment.

### 5.1 Limitation

Although this study will optimize certain shortcomings of the retrieved studies, it will still have limitations.

First, we excluded children with common comorbidities (ADHD, OCD, etc.) to avoid confounding. Psychiatric comorbidities are well-recognized as highly prevalent in tic disorders. Therefore, our findings apply primarily to the subgroup without these comorbidities; generalization to comorbid populations may overestimate the treatment effect and requires separate studies. The present trial is a necessary first step to establish a clean efficacy signal, and future studies will include children with stable comorbidities.

Second, the 8-week treatment and 4-week follow-up are relatively short. Tic disorders, especially transient tic disorder, can show natural improvement over time. However, the randomized controlled design ensures that any such natural improvement would occur equally across all groups. Therefore, a statistically significant difference between either active dose group and the control group would still indicate a genuine treatment effect beyond natural fluctuation. The current design does not allow for meaningful conclusions about the durability of treatment responses. Longer-term follow-up is needed and will be pursued once funding is available.

Third, our eligibility was restricted to children with SDPWS, which accounts for nearly half of pediatric tic disorder cases. If the trial is positive, the findings directly support the efficacy of PABG in this largest TCM subgroup, allowing generalization to nearly half of children with TD. For children with other TCM patterns, the results cannot be directly extrapolated; however, clinicians may consider individualized use based on pattern differentiation, recognizing that efficacy in those groups has not been established by this trial and requires separate investigation.

Fourth, the active control contained 5% of the active herbal ingredients to maintain blinding. Thus, the design tests PABG against a low-dose active control rather than an inert placebo, which may bias efficacy estimates toward the null. Future studies with a fully inert placebo are warranted.

Fifth, the sample size is insufficient to definitively establish a dose–response gradient, but the trial will provide descriptive information on dose-related trends.

Sixth, all participants were recruited from a single institution that also developed PABG, which may introduce selection and allegiance biases (though blinding and independent monitoring reduce detection bias). As a dose-finding study, a single-center design is acceptable; however, future confirmatory trials should be multicenter and independent of the product developer.

Seventh, a study suggests that fetal sex hormone exposure may play a role in the development of tic disorders (11). This biological subtyping, analogous to TCM syndrome differentiation, could be considered in future research to screen for optimal treatment candidates.

Despite these limitations, this trial will provide high-level evidence from an active-controlled dose-gradient study of PABG in children with TD, enabling an exploratory assessment of the dose–response relationship. If positive, the results could support registration of this in-house preparation under China’s regulatory pathway.

### 5.2 Trial status

Protocol version: V2.1 (17 July 2025). Recruitment start date: 15 September 2025; Expected recruitment completion date: 31 December 2026. This study protocol was submitted prior to the completion of participant recruitment.

## Glossary

TD: Tic Disorder
PABG: Pediatric Anshen Bunao Granules
TCM: Traditional Chinese Medicine
YGTSS: Yale Global Tic Severity Scale
TCM-SRS: TCM Syndrome Rating Scale
C&A-GTS-QoL: Gilles de la Tourette Syndrome- Quality of Life Scale for Children and Adolescents
ECG: Electrocardiogram
ADHD: Attention Deficit Hyperactivity Disorder
OCB: Obsessive-Compulsive Behavior
OCD: Obsessive-Compulsive Disorder
DSM-5: Diagnostic and Statistical Manual of Mental Disorders, Fifth Edition
TTD: Transient Tic Disorder
CTD: Chronic Motor or Vocal Tic Disorder
TS: Tourette Syndrome
CSTC: Cortical- Striatal- Thalamic- Cortical
ESSTS: European Society for the Study of Tic Disorders
AAN: American Academy of Neurology
SDPWS: Spleen Deficiency with Phlegm Accumulation and Wind- Phlegm Disturbance Syndrome
FXR: Farnesoid X Receptor
GABA: Gamma-Aminobutyric Acid
NMPA: National Medical Products Administration
CACM: Chinese Association of Chinese Medicine
RCT: Randomized Controlled Trial
ICF: Informed Consent Form
MCID: Minimal Clinically Important Difference
PASS: Power Analysis and Sample Size Software
NCSS: Number Cruncher Statistical System
SAS: Statistical Analysis System
IWRS: Interactive Web Response System
eCRF: Electronic Case Report Form
SAP: Statistical Analysis Plan
CI: Confidence Interval
ANCOVA: Analysis of Covariance
CMH: Cochran- Mantel- Haenszel
MedDRA: Medical Dictionary for Regulatory Activities
WHODrug: World Health Organization Drug Dictionaries
GCP: Good Clinical Practice
SOPs: Standard Operating Procedures
EDC: Electronic Data Capture
DMP: Data Management Plan
CRO: Contract Research Organization
AE: Adverse Event
SAE: Serious Adverse Event
ITMCTR: International Traditional Medicine Clinical Trial Registry
ULN: Upper Limit of Normal
AST: Aspartate Aminotransferase
ALT: Alanine Aminotransferase
ALP: Alkaline Phosphatase
